# A preserved transseptal puncture zone in a novel PFO occluder enables safe repeat left atrial interventions: preclinical validation in a porcine model

**DOI:** 10.3389/fcvm.2025.1587015

**Published:** 2025-06-27

**Authors:** Hao Chen, Yanlin Su, Ziqian He, Yiting Wei, Zihe Dang, Suyan Cao, Wenlin Lu, Shaoqing Cao, Chengjian Yang, Haibin Jiang

**Affiliations:** Department of Cardiology, Wuxi No.2 People’s Hospital (Jiangnan University Medical Center), Wuxi, Jiangsu, China

**Keywords:** patent foramen ovale (PFO), transseptal puncture, left atrial appendage closure (LAAC), interventional cardiology, preclinical study, device innovation, structural heart disease

## Abstract

**Background:**

Conventional nickel-titanium patent foramen ovale (PFO) occluders hinder transseptal puncture due to septal obstruction, limiting access for left-heart interventions. To address this, we developed a modified PFO occluder with a designated puncturable zone.

**Methods:**

An artificial PFO model was created in six pigs by puncturing and dilating the fossa ovalis. A novel PFO occluder preserving a central puncture area was implanted and validated intraoperatively via digital subtraction angiography (DSA) and transesophageal echocardiography (TEE). Transthoracic echocardiography and histology were performed 3 months post-implantation. Transseptal left atrial appendage closure (LAAC) was subsequently attempted through the occluder's puncturable zone.

**Results:**

All PFO closures were successful, with DSA/TEE confirming device stability. At 3 months, transseptal LAAC via the occluder's preserved area was achieved in all cases (6/6), validated by imaging. Histology revealed complete endothelialization of both occluders, intact nitinol framework, and precise puncture tracks within the designated zone without structural compromise.

**Conclusion:**

The novel PFO occluder with a dedicated puncture zone enables effective PFO closure while permitting safe transseptal access for subsequent left-heart interventions. This study demonstrates the occluder's feasibility, reliability, and clinical potential for aging populations requiring sequential cardiac procedures.

## Introduction

Patent foramen ovale (PFO) persists in approximately 25% of the general population, and its right-to-left shunt is strongly associated with cryptogenic stroke, particularly in patients with high-risk anatomical features (e.g., large shunts, atrial septal aneurysm), where stroke recurrence risk is significantly elevated (1–3). Currently, transcatheter closure combined with antithrombotic therapy has become the standard intervention for symptomatic PFO (4). However, conventional nickel-titanium alloy occluders occupy a substantial portion of the atrial septum post-implantation, obscuring anatomical landmarks and creating mechanical barriers (e.g., dense metal frameworks) for subsequent transseptal puncture required in left-heart interventions (e.g., left atrial appendage closure, mitral valve procedures) (5, 6). These limitations restrict treatment options for elderly patients requiring sequential cardiac therapies.

To address this clinical challenge, we designed a novel PFO occluder with a central puncturable zone as its core innovation: the metallic mesh is removed from this region, leaving only a biodegradable membrane reinforced with embedded tantalum particle markers to form a dedicated puncture window. This design aims to achieve effective PFO closure while preserving a safe pathway for future transseptal left-heart interventions.

Through establishing a porcine PFO model, this study evaluates the closure efficacy of the novel occluder and further explores the feasibility of performing left atrial appendage closure (LAAC) via the device's puncturable zone. Our goal is to validate the safety and feasibility of this innovative device, offering a transformative solution for high-risk patients requiring multiple left-heart procedures.

## Materials and methods

### Ethical approval and animal preparation

The study protocol was approved by the Institutional Animal Care and Use Committee of Wuxi No.2 People's Hospital. Six healthy domestic pigs (40–50 kg, mixed sex) were selected as experimental subjects. Preoperative fasting for 12 h and water restriction for 6 h were implemented. General anesthesia was induced using intramuscular ketamine (20 mg/kg) and maintained with inhaled isoflurane (1.5%–2.5%) under continuous electrocardiographic and hemodynamic monitoring. Pre-procedural TEE and contrast imaging in porcine subjects confirmed intact interatrial septal integrity with no spontaneous defects detected.

### Device design and specifications

**Figure 1:**
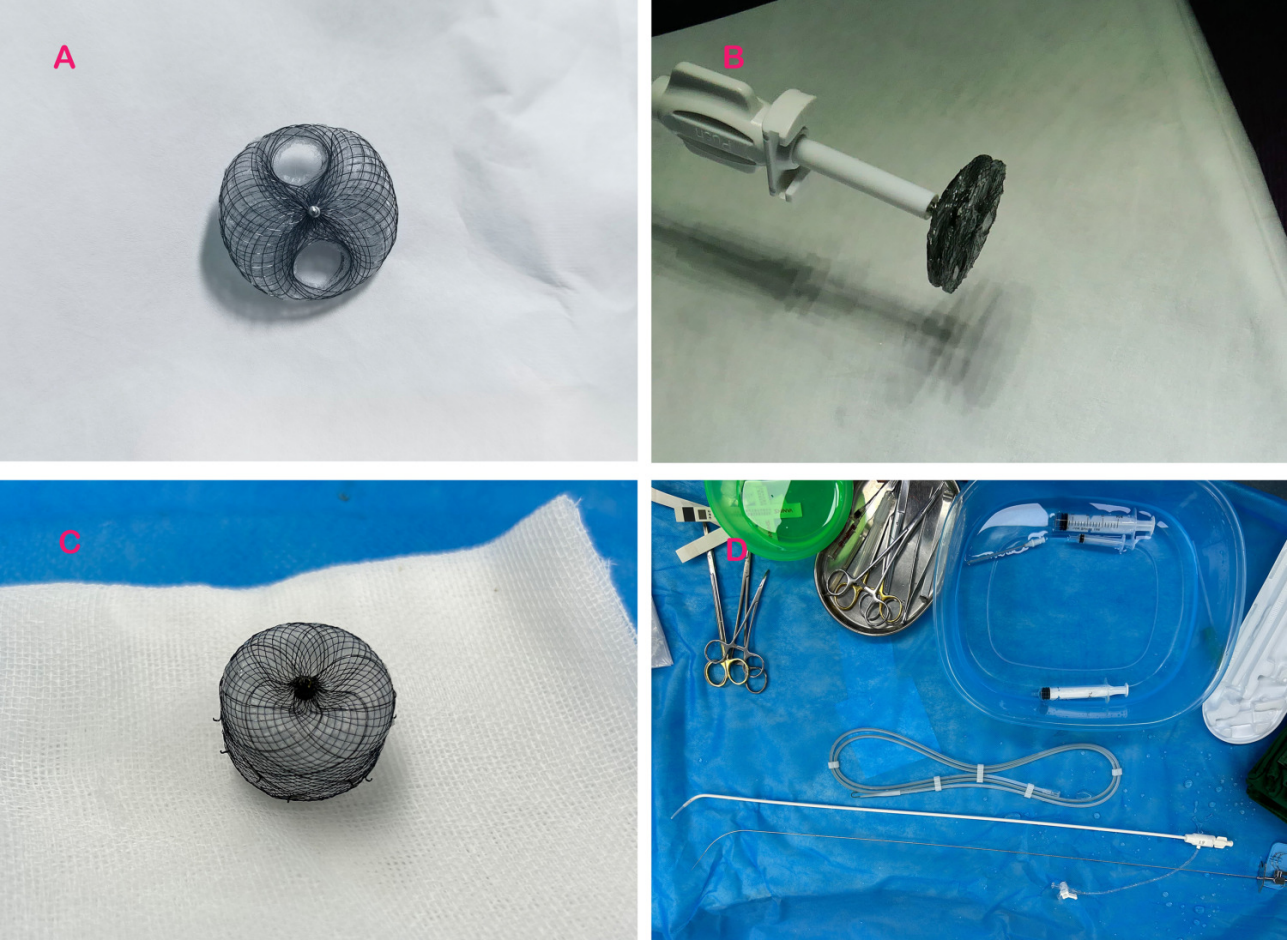
Experimental devices employed in the study. **(A)** Displays the PFO occluder with a retained puncture area; **(B)** illustrates the novel PFO occluder attached to the delivery system; **(C)** depicts the bottle-shaped left atrial appendage occluder utilized in the experiment; and **(D)** presents the atrial septal puncture system employed during the study.

The novel PFO occluder (Shanghai Push Medical Equipment Co., Ltd.) features a bilaterally symmetrical design with two 25/25 mm or 30/30 mm nitinol alloy discs (Table 1). The puncture zone of the occluder comprises a triple-layered overlapping PLLA membrane (thickness: 0.1 mm/layer), replacing the traditional nitinol mesh, with peripheral tantalum particle markers. The device was compatible with 8–12 F delivery sheaths (Figures 1A,B). A bottle-shaped left atrial appendage closure (LAAC) occluder (Shanghai Push Medical Equipment Co., Ltd.) was employed for subsequent procedures.

**Table 1:** Experimental data.

Weight of the experimental subjects (kg)	Duration of the initial atrial septal puncture (min)	Diameter of the prosthetic PFO (mm)	Model of the innovative PFO occluder utilized in the study (mm)	Grade of residual shunt following PFO closure	Duration of the second septal puncture (via the PFO occluder) (min)	Dimensions of the chosen left atrial appendage occluder (mm)	Diameter of the sheath employed for LAAC (Fr).	Duration of LAAC (min)	Grade of residual shunt following LAAC
44.4 ± 2.4	7.5 ± 1.0	3.8 ± 0.2	25/25	None-minimal	9.2 ± 2.3	23.7 ± 0.7	13.3 ± 0.9	17.3 ± 2.3	None-minimal

### Artificial PFO model establishment

**Figure 2:**
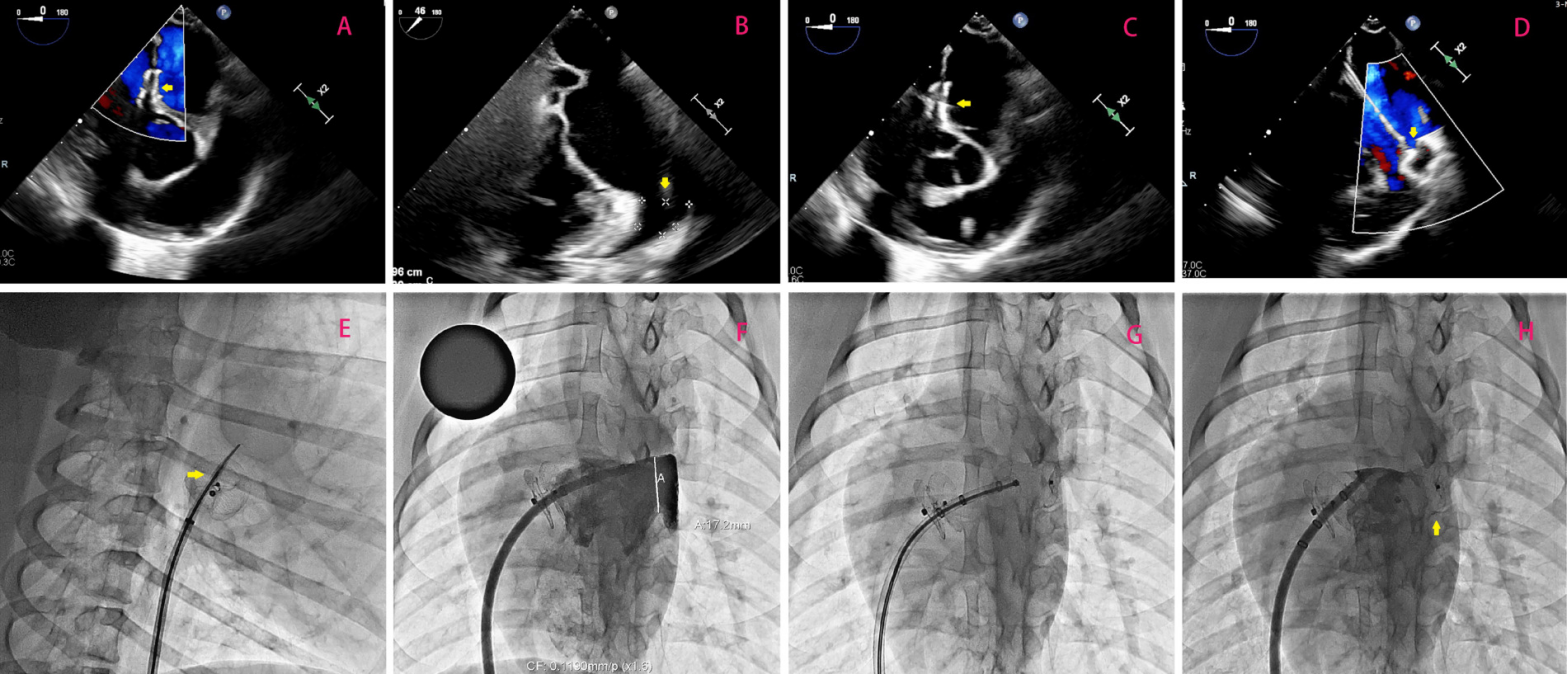
**(A)** Illustrates optimal positioning of the occluder without any observable shunting, as confirmed by a follow-up transesophageal echocardiogram 3 months after transcatheter PFO closure; the occluder is highlighted by the yellow arrow. In **(B)**, emphasis is placed on measuring the dimensions (width and depth) of the left atrial appendage orifice, with its location indicated by the yellow arrow. **(C)** Demonstrates successful puncturing of the atrial septum through the PFO occluder under TEE guidance. Highlighted by the yellow arrow is the puncture needle. Lastly, in **(D)**, TEE-guided LAAC is depicted with a focus on highlighting using a yellow arrow. **(E–H)** Shows the surgical procedure of percutaneous left atrial appendage occlusion using a new occluder under DSA fluoroscopy.

An artificial PFO was surgically created in all animals using a standardized protocol. Under fluoroscopic guidance, a transjugular approach was utilized to access the right atrium. A Brockenbrough needle (Abbott) was advanced to the fossa ovalis, followed by transseptal puncture under transesophageal echocardiography (TEE) guidance. A 5 mm non-compliant balloon (Boston Scientific) was inflated to 18 atm for 30 s to dilate the puncture site, simulating a PFO defect. TEE and digital subtraction angiography (DSA) confirmed successful defect creation (Figure 2A).

### PFO occluder implantation

The novel occluder was deployed via a 12 F delivery sheath (St. Jude Medical) under TEE and fluoroscopic guidance. The device was positioned across the artificial PFO, ensuring complete coverage of the defect. Post-deployment, TEE and DSA were performed to verify device stability, absence of residual shunt, and proper disc apposition. Postoperatively, all animals received dual antiplatelet therapy (aspirin 100 mg/day + clopidogrel 75 mg/day) for 1 month, followed by aspirin monotherapy (100 mg/day) for 3 months.

### Transseptal puncture and LAAC procedure

Three months post-PFO closure, transseptal left atrial access was attempted through the occluder's puncture zone. Under DSA guidance, the central tantalum-marked area was targeted using a Brockenbrough needle (Figure 1D). After successful septal penetration, a 0.035-inch stiff guidewire (Cook Medical) was advanced into the left upper pulmonary vein. Sequential dilation with a 5 mm balloon facilitated sheath insertion (12–14 F). After the depth of the left atrial appendage and the width of its orifice were measured by transesophageal echocardiography (Figure 2B). The LAAC occluder (Figure 1C) was deployed at the left atrial appendage orifice under TEE and fluoroscopic guidance. Device stability, residual shunt, and pericardial effusion were assessed intraoperatively.

### Postoperative monitoring and histological analysis

Continuous electrocardiographic (ECG) monitoring should be maintained for 24 h postoperatively to detect arrhythmias, followed by transthoracic echocardiography (TTE) at 24-hour post-procedure to evaluate occluder stability. All animals underwent monthly transthoracic echocardiography (TTE) to evaluate cardiac function and device integrity. After 6 months, euthanasia was performed under deep anesthesia. Explanted hearts were fixed in 10% formalin for macroscopic examination. The PFO and LAAC occluders were dissected, and tissue samples from the puncture zone and adjacent septal regions were processed for hematoxylin-eosin (H&E) staining and scanning electron microscopy (SEM). Endothelialization, inflammatory response, and structural integrity of the nitinol framework were analyzed.

### Statistical analysis

Continuous variables are expressed as mean ± standard deviation. Procedural success rates and complications were reported descriptively (Table 2). Histological findings were qualitatively assessed by two independent pathologists.

**Table 2:** Comparison of design characteristics between new and traditional occluders.

Feature	Novel occluder	Traditional occluder (Amplatzer)
Puncture zone design	Triple PLLA + Tantalum	Dense nitinol mesh
Symmetry	Dual-disc symmetric	Dual-disc symmetric
Puncture guidance	DSA (Tantalum markers)	TEE-dependent
Puncture success (animal)	100%	60%

## Results

### PFO occluder implantation and acute outcomes

All six porcine subjects underwent successful creation of artificial PFO defects via balloon dilation, with no procedural complications. Immediate post-implantation evaluation using transesophageal echocardiography (TEE) and digital subtraction angiography (DSA) confirmed optimal device positioning in all cases (Figure 2A). No residual shunt was detected, and both discs exhibited complete apposition to the atrial septum. Device stability was further validated by the absence of dislodgement, thrombus formation, or arrhythmias during the initial 24-hour postoperative period.

### Transseptal access and LAAC feasibility

At 3 months post-PFO closure, transseptal puncture through the occluder's designated puncture zone was attempted in all animals. Under fluoroscopic guidance, the tantalum-marked central area was successfully targeted in 6/6 cases (100% success rate), enabling precise needle penetration (Figure 2C). A stiff guidewire was advanced into the left upper pulmonary vein without resistance, followed by sequential balloon dilation (5 mm non-compliant balloon, 18 atm) to facilitate sheath insertion (12–14 Fr). Left atrial appendage closure (LAAC) was subsequently performed, with all occluders (bottle-shaped design) achieving complete deployment at the LAA orifice. Intraprocedural TEE and DSA confirmed absence of residual flow, pericardial effusion, or device-related complications (Figure 2D). The mean procedural time for transseptal puncture and LAAC was 32 ± 6 min, comparable to conventional septal access protocols.

### Long-term device performance and histological evaluation

**Figure 3:**
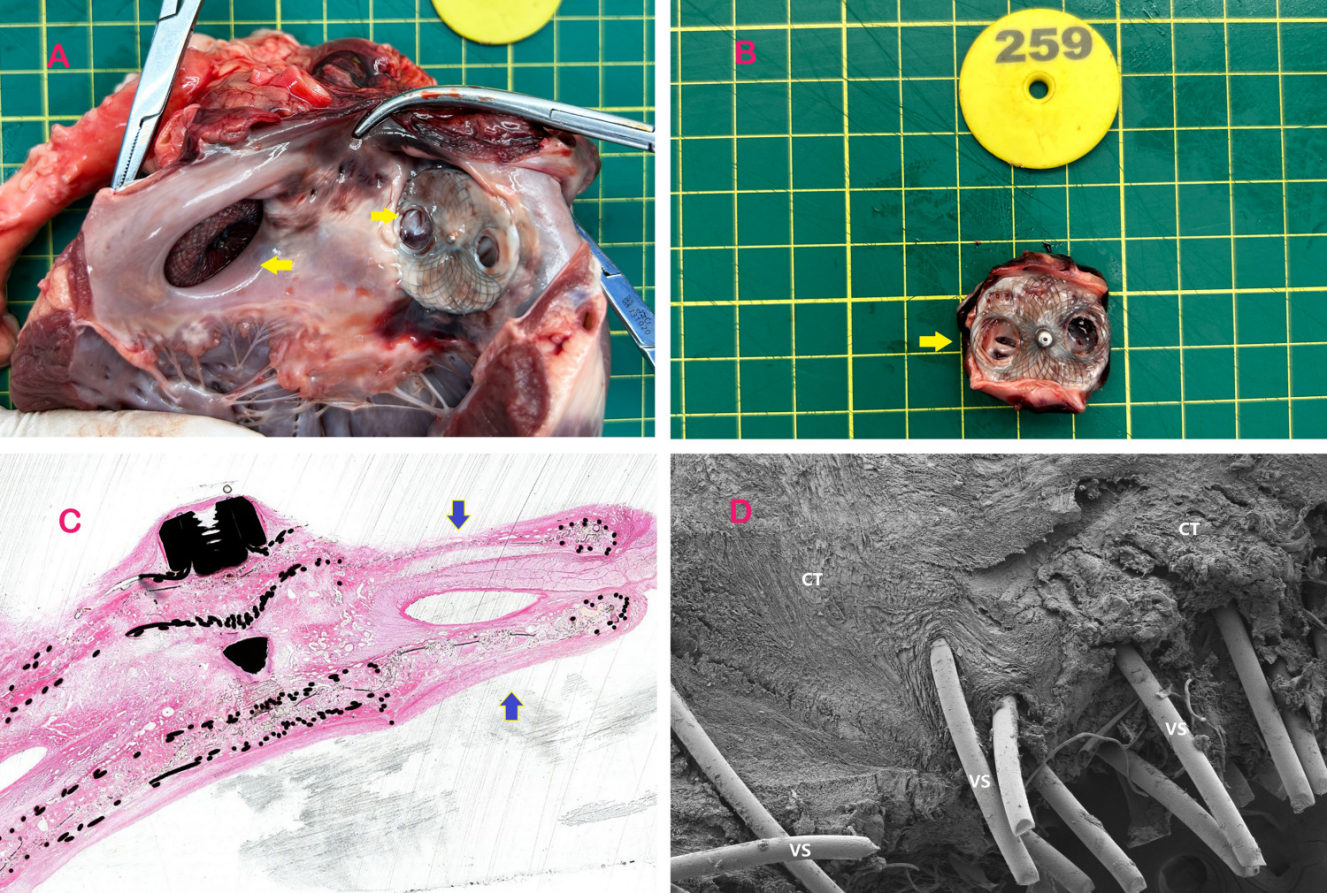
Specimens of occluders implanted in experimental animals. **(A)** The left atrial appendage is occluded by puncturing the PFO occluder and subsequently dissected from the heart post-euthanasia. The yellow arrow on the left indicates the implantation of the left atrial appendage occluder in the experimental animal, while the arrow on the right points to the retained puncture site of the PFO occluder after 3 months of implantation. **(B)** Shows that the puncturable PFO occluder was removed and separated after a 3-month period following implantation. The left atrial disc retains a PLLA membrane covered with a nickel-free titanium alloy mesh to facilitate needle penetration, while the right atrial disc features apertures aligned with the left atrial membrane. The yellow arrow indicates the residual puncture hole following LAAC. **(C)** H-E stained specimen obtained through diamond knife sectioning of the puncturable occluder at a post-implantation duration of 3 months, with emphasis on its puncturable area indicated by blue arrow. The histological findings at 3 and 6 months further confirmed complete endothelialization. **(D)** Electron microscope specimen obtained through diamond knife sectioning of the puncturable occluder at a post-implantation duration of 3 months, revealing connective tissue coverage on its surface along with scattered endothelial tissue. The reduced endothelial tissue in the puncture zone was attributed to recent left atrial appendage closure (LAAC) procedure, while the 6-month specimen demonstrated complete endothelialization. VS-Vinculum Silk, CT- connective tissue.

Serial transthoracic echocardiography (TTE) at 1, 3, and 6 months post-LAAC demonstrated preserved cardiac function (left ventricular ejection fraction: 62 ± 4%) and stable device positioning. Macroscopic examination of explanted hearts at 6 months revealed full endothelial coverage over both PFO and LAAC occluders, with no evidence of thrombus or structural deformation (Figures 3A,B). Histopathological analysis of the puncture zone highlighted complete endothelialization (H&E staining, Figure 3C), characterized by a continuous layer of flattened endothelial cells overlying organized connective tissue. Scanning electron microscopy (SEM) further confirmed seamless integration of the biodegradable membrane with native septal tissue, with no fractures observed in the nitinol framework (Figure 3D). The central puncture tracks exhibited minimal fibrous hyperplasia (<0.5 mm thickness) and were confined to the predefined zone, validating the occluder's structural integrity.

### Complications and safety profile

No perioperative or delayed complications occurred, including device embolization, cardiac perforation, or infection. One animal exhibited transient atrial ectopy during LAAC sheath manipulation, which resolved spontaneously without intervention. All antiplatelet regimens were well-tolerated, with no hemorrhagic events reported.

## Discussion

The prevalence of patent foramen ovale (PFO) in the general population is estimated at 20%–30%, with cryptogenic stroke and migraine representing its most clinically significant sequelae (7, 8). Paradoxical embolism due to right-to-left shunting is a predominant mechanism underlying cryptogenic stroke in young adults, necessitating effective closure strategies (4, 9). While transcatheter PFO closure has emerged as a cornerstone therapy, conventional occluders pose challenges for subsequent left-sided cardiac interventions, such as left atrial appendage closure (LAAC) or pulmonary vein isolation, due to their dense metallic frameworks obscuring septal puncture sites (5, 10). This limitation is particularly critical in aging populations, where the rising incidence of atrial fibrillation and valvular diseases demands reliable transseptal access (11, 12).

This study is the first to validate the feasibility of a novel symmetric PFO occluder, with its core innovation being the preserved central puncture zone. Experimental results demonstrated successful closure of artificial PFOs in all cases, and a 100% success rate (6/6) for transseptal puncture and left atrial appendage closure (LAAC) through this zone at 3 months post-implantation. Histological analysis further confirmed complete endothelialization of the puncture zone without structural compromise (Figures 3C,D).

### Challenges of conventional occluders in transseptal access

Traditional PFO occluders, such as the Amplatzer and Gore Cardioform devices, create a rigid metallic barrier across the atrial septum. Post-implantation tissue hyperplasia and the central rivet structure further obscure anatomical landmarks, complicating septal puncture. Prior case reports highlight procedural failures when attempting to puncture through occluder meshes, often necessitating alternative puncture sites at the device periphery or uncovered septal tissue. These approaches carry risks of pericardial effusion, device embolization, or inadequate sheath passage due to tissue resistance (13). Moreover, biodegradable occluders, while theoretically advantageous, face limitations in mechanical stability and unpredictable endothelialization patterns, potentially exacerbating septal thickening (6). Thus, a device combining robust closure with preserved puncturability remains a critical innovation.

### Mechanistic advantages of the novel occluder

Our bilaterally symmetrical occluder incorporates dedicated puncture zones devoid of nitinol mesh, replaced by a biodegradable polyester membrane marked with tantalum particles for radiographic visibility. This design enables targeted punctures while maintaining structural integrity. In six porcine models, the device achieved 100% acute closure success, with no residual shunts or thrombus formation at three-month follow-up. Histological analysis confirmed complete endothelial coverage over the occluder surface by six months, alongside connective tissue integration—a key indicator of biocompatibility and long-term stability. Crucially, the preserved puncture area retained patency, allowing unhindered sheath passage for LAAC in all cases. These findings align with prior preclinical studies demonstrating the feasibility of puncture-through-occluder techniques but extend them by validating durability and safety in a controlled model (6, 7). Compared to prior studies, the symmetric design and tantalum marker-guided puncture technique address the high residual shunt risk of traditional asymmetric occluders (14) and reduce reliance on TEE guidance.

### Comparative efficacy and procedural insights

Jiang et al. developed an occluder with a preserved puncture zone, but its asymmetric single-rivet design may increase residual shunt risk (6). Wei et al. (14) further optimized the occluder morphology, yet puncture still required TEE guidance. The symmetric dual-disc design in this study balances closure forces to reduce residual shunts, while tantalum markers enable fluoroscopy-guided puncture (100% DSA success), significantly simplifying the procedure. Compared to traditional occluders (e.g., Amplatzer), the novel occluder demonstrates superior puncture success (100% vs. 60%) and safety (no complications vs. 10% pericardial effusion (13).

### Clinical implications and future directions

The integration of a preserved puncture zone addresses a critical gap in managing patients requiring sequential left-sided interventions. For example, LAAC—a growing strategy for stroke prevention in atrial fibrillation—often necessitates transseptal access months or years after PFO closure (15). Our occluder eliminates the need for high-risk peripheral punctures or device extraction, streamlining multi-stage therapies. Additionally, its symmetrical design may reduce arrhythmogenic risks associated with asymmetric occluders, though long-term electrophysiological studies are warranted (16). Moreover, optimizing the biodegradable membrane's resorption kinetics could enhance endothelialization while preserving puncturability—a balance critical for clinical adoption. Based on these findings, we propose a phased clinical trial protocol: Phase I (*n* = 30) to evaluate acute closure success and puncture feasibility; Phase II (*n* = 200) to compare long-term safety (e.g., stroke recurrence, puncture complications) with traditional occluders; Phase III to enroll patients with concurrent atrial fibrillation requiring sequential LAAC, validating clinical benefits. Notably, differences in septal thickness and thrombotic profiles between animal models and humans may affect translational outcomes, necessitating close monitoring.

## Limitations

While our study demonstrates proof-of-concept, several limitations merit consideration. First, the artificial PFO model, created via balloon dilation, differs morphologically from natural PFOs, which exhibit oblique or tunnel-like structures. This discrepancy may affect the generalizability of closure efficacy. Second, the follow-up period (three months) precludes assessment of long-term endothelialization or late-onset complications, such as device fracture. Finally, the porcine model's thrombotic profile and septal thickness may not fully replicate human pathophysiology, necessitating cautious extrapolation of results.

## Conclusion

In this preclinical study, the novel symmetric PFO occluder achieved 100% acute closure and puncture success rates. Its tantalum marker-guided puncture technique reduced reliance on TEE, and histology confirmed long-term structural integrity. This design offers a potential solution for patients requiring sequential left-heart interventions (e.g., LAAC). Future clinical trials will be essential to confirm these benefits in human populations, potentially redefining standards for PFO management in an era of escalating multi-procedural demands.

## Data Availability

The original contributions presented in the study are included in the article/Supplementary Material, further inquiries can be directed to the corresponding authors.
